# Beyond Treatment: Prevalence, Predictors, and Changes in Anxiety and Depression Among Parents of Childhood Cancer Survivors

**DOI:** 10.1002/pon.70115

**Published:** 2025-03-02

**Authors:** Hannah Kurz, Verena Paul, Mona L. Nasse, Konstantin A. Krauth, Daniela Kandels, Stefan Rutkowski, Gabriele Escherich, Laura Inhestern, Corinna Bergelt

**Affiliations:** ^1^ Department of Pediatrics University Medical Center Hamburg‐Eppendorf Hamburg Germany; ^2^ Department of Medical Psychology University Medical Center Hamburg‐Eppendorf Hamburg Germany; ^3^ Department of Pediatrics, Pediatric Hematology and Oncology Klinik Bad Oexen Bad Oexen Germany; ^4^ Medical Faculty Swabian Children's Cancer Center University of Augsburg Augsburg Germany; ^5^ Department of Pediatric Hematology and Oncology University Medical Center Hamburg‐Eppendorf Hamburg Germany; ^6^ Partner Site Hamburg of the German Center for Child and Adolescent Health (Deutsches Zentrum für Kinder‐ und Jugendmedizin, DZKJ) Hamburg Germany; ^7^ Department of Medical Psychology University Medical Center Greifswald Greifswald Germany

**Keywords:** anxiety, cancer, cancer survivors, central nervous system neoplasms, depression, leukemia, oncology, parents, psycho‐oncology, risk factors

## Abstract

**Background:**

Childhood cancer remains a significant psychological burden for parents. Even after end of treatment, parents of childhood cancer survivors remain at high risk of developing anxiety and depression. However, knowledge about the prevalence and changes of these conditions post‐treatment is limited.

**Aims:**

This study aimed to assess the proportion of parents exhibiting clinically relevant symptoms of anxiety and/or depression, explore gender differences, examine factors associated with these conditions and their longitudinal data.

**Methods:**

Five hundred and sixteen parents of childhood cancer survivors (aged 0–17 years at diagnosis of leukemia or central nervous system tumors) were evaluated after treatment and again 12–18 months later. Anxiety (GAD‐7) and depression (PHQ‐9) symptoms were assessed. Generalized linear mixed models were used to analyze factors influencing these conditions and their changes.

**Results:**

At baseline, 30% of parents reported clinically relevant depression, and 34% reported anxiety, both decreasing to 16% at follow‐up. Mothers reported higher anxiety and depression scores, with more meeting clinically relevant thresholds. Low family functioning, psychotherapy, physical illness, and a recent diagnosis were significant predictors of both conditions. Additional predictors for depression included unemployment, single‐parent status, and fear of progression, while female gender was a predictor for anxiety in the final model. Greater symptom improvements were associated with higher baseline symptoms, whereas longer time since diagnosis was linked to less improvement.

**Conclusion:**

Anxiety and depression represent significant burdens for parents of childhood cancer survivors, with several modifiable risk factors identified. Targeted psychosocial support, early screening, and tailored interventions may reduce distress and improve family well‐being.

**Abbreviations**
CCS: childhood cancer survivors
CNS: central nervous system
EOT: end of treatment
FoP: fear of progression
GLMM: generalized mixed model
ICC: intraclass correlation coefficient
SES: socioeconomic status

## 1 Background

Approximately 1 out of 300 newborns in Europe will develop cancer before reaching the age of 20 [1]. Although childhood cancer is rare, affecting fewer than 1 in 2000 children, it remains the leading cause of disease‐related death among children and adolescents in Europe [2]. The 5‐year survival rates for pediatric cancer diseases in Europe have dramatically increased, still approximately 20% of children diagnosed with cancer in developed countries will not survive the disease [3]. Notably, nearly 40% of cancer deaths in children are due to central nervous system (CNS) tumors [2, 3]. Despite advancements in diagnostics, treatment options, and survival rates, a child's cancer diagnosis remains an emotionally stressful event with potential long‐lasting consequences for the entire family [4, 5, 6]. The diagnosis of childhood cancer has been described as one of the most intense, disruptive, and enduring experiences that parents can have [4, 7]. Hospital admissions, not being able to work anymore, worries about the survival of the affected child, fear of progression as well as worries about neglecting siblings and relationship problems might suddenly predominate everyday family life [4, 6, 7, 8]. Whereas children may adapt quickly to new situations, coping with a child's illness—both during and after the end of treatment (EOT)—can contribute to the development of anxiety and depressive disorders in parents, even years after treatment [8, 9, 10]. These conditions might have serious consequences, not only for the individual, but also for the society.

According to a recent meta‐analysis, the prevalence of anxiety and depression among parents of children with cancer varies widely, ranging from 5% to 65% for anxiety and 7%–91% for depression [11]. This wide range might reflect high methodological heterogeneity among different studies, including measurement tools and the parent being surveyed, as mothers often report higher levels of distress compared to fathers [5, 12]. Additionally, multiple studies indicate that parents experience higher levels of depression and anxiety closer to diagnosis [10, 11, 13]. Overall, little is known about anxiety and depressive symptoms in parents of childhood cancer survivors (CCS), particularly in the post‐treatment phase. At that point, families are discharged from structured treatment plans and return to daily life. Parents may experience feelings of emptiness and exhaustion, which can contribute to psychological distress [14, 15]. Addressing this transition period is crucial to identify parents at greatest risk for maladjustment and in need of additional support from healthcare providers. Moreover, there is limited knowledge about how these conditions evolve during the aftercare period.

Hence, this study aimed to assess anxiety and depression symptoms among parents of CCS at two critical time points: immediately after the end of acute treatment (EOT) and 12–18 months later during aftercare. Our research objectives were: (1) to examine the prevalence of clinically relevant symptoms after EOT and during aftercare, (2) to identify parent‐, family‐, and patient‐related factors associated with these symptoms after EOT, and (3) to explore factors related to symptom changes during the aftercare period. Given the limited data available, the selection of potentially associated factors followed a rather explorative approach and was based on prior research on parental distress in childhood cancer and on our clinical experience [4, 5, 7, 8, 9]. By identifying parents at higher risk for psychological distress, the study aimed to provide a deeper understanding of how parental mental health evolves during aftercare. These insights may contribute to the development of targeted interventions aimed at reducing the negative impact of these conditions and improving the well‐being of families affected by pediatric cancer.

Based on literature, we hypothesized that:

1. Levels of anxiety and depression in parents of CCS are higher than in normative samples.
2. Mothers report higher levels of anxiety and depression compared to fathers.
3. Longer time since treatment completion is associated with lower levels of anxiety and depression.
4. Baseline levels of anxiety and depression, as well as changes in these symptoms from baseline to follow‐up, are predicted by parent‐, family‐, and patient‐related variables.

## 2 Methods

### 2.1 Design

This study is a secondary analysis of data from a prospective observational study with a longitudinal mixed method design [14]. The overall study was approved by the Ethics Committee of the Medical Chamber of Hamburg (number PV5277) and has been described in a study protocol [14].

### 2.2 Participants and Procedure

The overall study focused on leukemia and brain tumors, the most common childhood cancer diagnoses in Germany [16]. Biological parents and other caregivers of children diagnosed before age 18 were included. Participants completed standardized self‐report questionnaires either at the end of acute treatment or the beginning of rehabilitation (baseline) and at a 12‐ to 18‐months follow‐up. Exclusion criteria were insufficient German language skills, severe cognitive impairments, or too high physical/mental burden (self‐assessed or determined by healthcare providers).

Data were collected in Germany between July 2016 and December 2020 through two recruitment methods: via national pediatric cancer study registries (COALL 08‐09 [NCT0122833], I‐HIT‐MED [NCT02417324], and SIOP‐LGG 2004 [NCT00276640]) or through a cooperating rehabilitation clinic. In both cases, parents were informed by healthcare providers and received study materials upon willingness to participate. Detailed information can be found in the study protocol [14].

### 2.3 Measures

#### 2.3.1 Sociodemographic and Medical Data

Sociodemographic (e.g., gender) and medical (e.g., diagnosis type) data were assessed by self‐developed questionnaires. Based on the recruitment method, the diagnosis and time since diagnosis were reported either by physicians at the rehabilitation clinic or by parents. All other variables were self‐reported by the parents. The socioeconomic status (SES) was calculated by using the Winkler Index, which is based on three variables: income, education and occupation [17].

#### 2.3.2 Anxiety

Parental anxiety symptoms were assessed by the Generalized Anxiety Disorder Scale (GAD‐7). The 7‐item questionnaire is based on the Diagnostic and Statistical Manual of Mental Disorders (DSM‐IV) for generalized anxiety disorders [18]. Response options range from 0 (not at all) to 3 (nearly every day) on a 4‐point Likert scale. Sum scores ≥ 10 were used as cut‐off in the present analyses to indicate clinically relevant anxiety symptoms [18]. The GAD‐7 has proved to be reliable and valid [18]. Cronbach's alpha in our sample was 0.86.

#### 2.3.3 Depression

Parental depressive symptoms were assessed by the PHQ‐9, a 9‐item depression scale of the Patient Health Questionnaire [19]. It rates depression severity based on self‐reported items on a 4‐point Likert scale scoring from 0 (not at all) to 3 (nearly every day). Clinically relevant depressive symptoms were defined by a sum score of ≥ 10 with a sensitivity of 88% and a specificity of 88% for major depression [19]. The PHQ‐9 has proved to be a reliable and valid questionnaire [19]. Cronbach's alpha in our sample was 0.85.

#### 2.3.4 Family Functioning

Family functioning was assessed by the general functioning scale of the Family Assessment Device (FAD‐GF) [20]. The FAD is based on the McMaster Model of Family Functioning (MMFF)—a clinical orientated conceptualization of family functioning. The FAD‐GF sub scale comprises 12 items ranking on a 4‐point Likert scale from 1 (strongly agree) to 4 (strongly disagree). The FAD‐GF includes different features of family functioning such as communication skills. Higher scores indicate lower family functioning. The FAD‐GF is proven to be a reliable and valid questionnaire [20]. Cronbach's alpha in our sample was 0.86.

#### 2.3.5 Fear of Progression

Parental fear of progression (FoP) was assessed by the Fear of Progression Questionnaire‐Short Form for Parents (FoP‐Q‐SF/PR) [21]. This questionnaire is a modified version of the FoP‐Q‐SF designed to assess fear of further disease progression in adult patients. The FOP‐Q‐SF/PR sub scale comprises 12 items ranking on a 5‐point Likert scale from 1 (never) to 5 (very often). The FoP‐Q‐SF/PR generates a sum score, with higher scores reflecting higher levels of FoP. As in an earlier study on FoP in parents of childhood cancer patients, scores ≥ 34 were used as a cut‐off for dysfunctional FoP [22]. The FoP‐Q‐SF/PR has demonstrated reliability and validity [21]. Cronbach's alpha in our sample was 0.86.

### 2.4 Statistical Analysis

To examine the sample, descriptive statistics (frequencies, means, standard deviations, medians, and ranges) were calculated. Differences in psychological measures between mothers and fathers (GAD‐7 for anxiety, PHQ‐9 for depression, FAD‐GF for family functioning, and FoP‐Q‐SF/PR for FoP) were analyzed using unpaired *t*‐tests and Chi^2^ tests.

We examined whether anxiety and depression at the EOT (*T*1) and changes in symptom levels (Δ*T* = *T*2 − *T*1) during the aftercare period could be predicted by baseline variables categorized into parent‐, family‐, and patient‐related factors. Variable selection was based on prior research and clinical experience, considering both established risk factors (e.g., unemployment, diagnosis type) and general correlates of psychological distress (e.g., fear of progression) [4, 6, 7, 8, 9].

To account for the hierarchical data structure, with two family members assessed, generalized linear mixed models (GLMM) were used to identify predictors, allowing for both fixed and random effects [23]. Two models were constructed for both anxiety and depression: one predicting baseline symptoms and one predicting symptom changes. In the longitudinal analysis, the GLMM outcomes were represented by the difference in sum scores between *T*2 and *T*1 (Δ*T* = *T*2 − *T*1). A two‐step approach was applied to all outcomes. Given the exploratory nature of the study, univariate analyses were conducted first, and correlation tables were calculated to include all relevant variables. Variables were grouped into parent‐related factors (e.g., age, gender, single parent status, unemployment), patient‐related factors (e.g., age, gender, diagnosis type, time since diagnosis), and family‐related factors (family functioning). FoP was included as a parent‐related factor in the depression models but excluded from the anxiety models to avoid using a variable closely linked to anxiety.

Variables with significant correlations (*p* < 0.05) were selected for further analysis. Although unemployment did not show significant correlations in the preliminary analysis, it was included in the anxiety models (both baseline and longitudinal) due to its relevance in the literature [24, 25]. Patient‐related factors (e.g., children's gender and age, diagnosis type, and time since diagnosis) were included in all four models based on their clinical importance, regardless of statistical significance in the correlation analyses. To avoid multicollinearity, variables with high correlations (*r* ≥ 0.7) were excluded, retaining diagnosis‐related variables. “Patient's age at baseline” was excluded, and “age at diagnosis” was included.

In the second step, GLMMs were conducted to identify predictors for each of the four outcomes. For the baseline analyses, predictors were selected as described above. In the longitudinal analyses, additional predictors included baseline levels of anxiety and depression (*T*1 scores) to account for initial symptom severity, and all significant predictors identified in the baseline models to ensure continuity and account for their relevance. COVID‐19 was added as a covariate because some follow‐up assessments occurred during the pandemic (after March 2020). Although COVID‐19 was not significantly correlated with the outcomes, it was retained due to its established association with anxiety and depression in the literature [26, 27, 28].

All independent variables were included as fixed factors, and family affiliation was treated as a random effect to account for inter‐individual variability. Dummy coding was used where necessary. A step‐down stepwise procedure was applied to identify the most relevant predictors by removing non‐significant variables, ensuring that the final models contained fewer than 10 predictors. Given the exploratory nature of this study, this approach improves model interpretability without compromising explanatory power [23]. Missing values were replaced by the individual's mean, provided that < 30% of data were missing for a given scale. An alpha level of 0.05 was used for all analyses, which were conducted using IBM SPSS Statistics 28.

## 3 Results

### 3.1 Sample Features

Eight hundred and ninety‐nine families were potentially eligible to be enrolled in this study. The initial participation rate was 35%, with 312 families actively participating in the survey. Among the 587 families that did not participate, 527 were identified via the national study registries. They did not participate because they either could not be informed by healthcare providers, met the exclusion criteria, or were not interested in participation. The remaining 60 families that were ultimately excluded from the study were recruited via the rehabilitation clinic. Exclusion reasons were insufficient German language skills (*n* = 14), cognitive limitations (*n* = 3), high physical and/or mental burden (*n* = 12), lack of interest (*n* = 21), or unspecified reasons (*n* = 10). Out of the initially participating 312 families, 7 were subsequently excluded from these analyses due to an inaccurate diagnosis (*n* = 2), missing consent forms (*n* = 2), incomplete questionnaires due to insufficient German language skills (*n* = 1), or because only the children completed the survey (*n* = 2).

Consequently, data from 305 families with 516 parents were analyzed in this study. Of these, 131 families were recruited through study registries, and 174 via the rehabilitation clinic. Among the participating families, 211 involved both parents. A total of 292 parents completed the 12‐ to 18‐months follow‐up. The proportion of mothers was higher than that of fathers at both measurement points. Table 1 presents the sociodemographic characteristics of 516 parents and the medical data of 305 CCS.

**TABLE 1:** Sociodemographic and medical characteristics of parents (*n* = 516) and childhood cancer survivors (*n* = 305).

Parents^g^	Total (*n* = 516)		Mothers (*n* = 299)		Fathers (*n* = 217)	
	*M*	SD/range	*M*	SD/range	*M*	SD/range
Age in years^a^	39.4	7.3/20–70	38.2	6.9/20–64	41.1	7.6/23–70

	*n*	%	*n*	%	*n*	%
Relationship status^b^						
Single	74	14.4	49	16.5	25	11.6
Married	396	77.2	216	72.7	180	83.3
Divorced	42	8.2	31	10.4	11	5.1
Widowed	1	0.2	1	0.3	0	0
Education in years of schooling^c^						
< 10 years	244	49.6	146	50.7	98	48.0
> 10 years	248	50.4	142	49.3	106	52.0
Employment status^d^						
Gainfully employed	365	77.8	168	57.3	197	92.5
Not gainfully employed	104	22.2	125	42.7	16	7.5
Socioeconomic status (SES)^e^ ^,^ ^h^						
Low	78	15.6	48	16.7	30	14.1
Medium	231	46.1	139	48.3	92	43.1
High	192	38.3	101	35.1	91	42.7

Patients	Total (*n* = 305)		Girls (*n* = 135)		Boys (*n* = 170)	
	*M*	SD/range	*M*	SD/range	*M*	SD/range
Age at baseline in years	7.3	4.3/1–18	6.6	4.0/1–17	7.4	4.5/1–18
Age at diagnosis in years	5.5	4.2/0–17	4.6	3.8/0–16	6.1	4.5/0–17
Time since diagnosis in months	22.1	21.8/5–178	22.2	22.5/5–178	22.1	21.3/5–152

	*n*	%	*n*	%	*n*	%
Number of siblings						
0	59	19.3	30	22.2	29	17.1
1–2	222	72.8	98	72.6	124	72.9
> 2	24	7.9	7	5.2	17	10
Cancer diagnosis						
Leukemia	189	62.0	88	65.2	101	59.4
CNS tumor	116	38.0	47	34.8	69	40.6
Other chronic diseases or impairments (e.g., epilepsy or hemiparesis)^f^	82	27.0	36	26.7	46	27.2

^a^ 2 missing.

^b^ 3 missing.

^c^ 25 missing.

^d^ 10 missing.

^e^ 15 missing.

^f^ 1 missing.

^g^ Summarizing parents and other caregivers.

^h^ According to the social strata index by Winkler [17].

### 3.2 Descriptive Data and Gender‐Based Differences

Mothers reported statistically significant higher depression scores (PHQ‐9) at both measurement points (Table 2). The overall sample had a mean depression score of *M* = 7.4 (SD 5.2) at baseline, with 30% of parents showing clinically relevant symptoms (sum score ≥ 10) [19, 20]. This proportion decreased to 16% at the 12‐ to 18‐months follow‐up.

**TABLE 2:** Descriptive statistics and gender‐based differences in self‐reported outcomes among parents of childhood cancer survivors post‐treatment (*n* = 516) and at follow‐up (*n* = 292).

	Total		Mothers		Fathers
Baseline	*n* = 516		*n* = 299		*n* = 217
Follow‐up	*n* = 292		*n* = 171		*n* = 121

Depression (PHQ‐9)	*M*	SD/range	*M*	SD/range	*M*	SD/range	*t*	*p*	Cohen's *d*
Sum scores									
Baseline^a^	7.4	5.2/0–25	8.2	5.2/0–25	6.2	4.9/0–23	4.43	< 0.001	0.40
Follow‐up	5.6	4.7/0–25	6.2	5.0/0–25	4.7	4.1/0–19	2.72	0.007	0.32

	*n*	%	*n*	%	*n*	%		*p*	*χ* ^2^
Depression cut off^e^									
Clinically relevant level									
Baseline^a^	154	30.1	109	36.7	45	20.9		< 0.001	14.75
Follow‐up	47	16.2	31	18.2	16	13.2		0.25	1.31

Anxiety (GAD‐7)	*M*	SD/range	*M*	SD/range	*M*	SD/range	*t*	*p*	Cohen's *d*
Sum scores									
Baseline^b^	8.0	4.9/0–21	8.8	4.9/0–21	6.8	4.7/0–21	4.72	< 0.001	0.42
Follow‐up	5.8	4.3/0–21	6.5	4.6/0–21	4.9	3.8/0–19	3.22	0.001	0.40

	*n*	%	*n*	%	*n*	%		*p*	*χ* ^2^
Anxiety cut‐off^e^									
Clinically relevant level									
Baseline^b^	172	33.7	123	41.7	49	22.8		< 0.001	19.88
Follow‐up	46	15.8	33	19.4	13	10.7		0.05	3.99

Family functioning (FAD‐GF)	*M*	SD/range	*M*	SD/range	*M*	SD/range	*t*	*p*	Cohen's *d*
Sum scores									
Baseline^c^	1.8	0.5/1–4	1.8	0.5/1–3.5	1.8	0.5/1–4	0.88	0.379	0.08
Follow‐up	1.8	0.5/1–3.6	1.8	0.5/1–3.6	1.8	0.5/1–3.3	−0.45	0.652	−0.05

Fear of progression (FoP‐Q‐SF/PR)	*M*		*M*		*M*		*t*	*p*	Cohen's *d*
Sum scores									
Baseline^d^	33.8		34.9		32.3		3.10	0.002	0.28
Follow‐up	31.1		32.4		29.1		2.91	0.004	0.35

	*n*	%	*n*	%	*n*	%		*p*	*χ* ^2^
Fear of progression cut‐off^f^									
Clinically relevant									
Baseline^d^	248	48.3	161	53.9	87	40.1		0.003	8.70
Follow‐up	114	39.1	77	45.3	37	30.6		0.011	6.42

^a^ 4 missing.

^b^ 6 missing.

^c^ 9 missing.

^d^ 3 missing.

^e^ Sum scores ≥ 10 were considered dysfunctional.

^f^ Sum scores ≥ 34 were considered dysfunctional.

Anxiety levels (GAD‐7) followed a similar pattern, with mothers reporting significantly higher scores at both measurements. The mean anxiety score for the overall sample at baseline was *M* = 8.0 (SD 4.9), with 34% of parents showing clinically relevant symptoms (sum score ≥ 10) [18]. This proportion decreased to 16% at the 12–18‐months follow‐up.

Mothers also reported higher Fear of Progression scores (FoP‐Q‐SF/PR) at both measurement points. No significant differences between mothers and fathers were found in family functioning (FAD‐GF).

### 3.3 Predictors of Anxiety and Depressive Symptoms After the EOT

After accounting for inter‐individual differences due to family affiliation, the GLMM results identified several significant predictors for depressive and anxiety symptoms at baseline (Table 3). Across both final models, worse family functioning, previous participation in psychotherapy, and a serious physical illness of the parent were associated with higher symptom levels. Additionally, a longer time since diagnosis was linked to lower depressive and anxiety symptoms. Patient‐related factors such as the child's type of cancer (leukemia vs. brain tumor) did not significantly predict symptom levels.

**TABLE 3:** Mixed model estimates for predicting anxiety and depression in parents (*n* = 516) of childhood cancer survivors post‐treatment.

(a) Predictors of depressive symptoms in parents post‐treatment						
Factor	Est.	SE	*t*	*p*	95% CI	
					Lower	Upper
Intercept	−5.404	13.899	−3.888	< 0.001	−8.138	−2.670
Fixed effects						
Parent‐related factors						
Unemployed^a^	1.142	0.5059	2258	**0.024**	0.148	2137
Fear of progression^d^	0.228	0.0233	9.787	**<** **0.001**	0.183	0.274
Participation in psychotherapy^a^	2.247	0.4486	5.010	**<** **0.001**	1.365	3.129
Serious physical illness^a^	1.557	0.6727	2.315	**0.021**	0.235	2.879
Single parent	1.450	0.7286	1.990	**0.047**	0.017	2.883
Family‐related factors						
Family functioning^b^	2.396	0.4368	5.485	**<** **0.001**	1.537	3.255
Patient‐related factors						
Age at diagnosis	−0.071	0.0495	−1.428	0.155	−0.168	0.027
Diagnosis (leukemia)^c^	0.830	0.4320	1.922	0.056	−0.021	1.682
Time since diagnosis	−0.023	0.0100	−2.265	**0.024**	−0.042	−0.003
Random effects						
Residual *σ* ^2^	14.300	1.806	7.916	< 0.001	11.164	18.317
ICC	0.119					

(b) Predictors of anxiety symptoms in parents post‐treatment						
Factor	Est.	SE	*t*	*p*	95% CI	
					Lower	Upper
Intercept	3.520	11.781	2.988	**0.003**	1.204	5.836
Fixed effects						
Parent‐related factors						
Female parent/caregiver	1.687	0.3756	4.491	**<** **0.001**	0.947	2.426
Socioeconomic status^e^	−0.095	0.0493	−1.930	0.054	−0.192	0.002
Serious physical illness^a^	1.660	0.6808	2.438	**0.015**	0.322	2.998
Participation in psychotherapy^a^	1.961	0.4437	4.418	**<** **0.001**	1.089	2.833
Family‐related factors						
Family functioning^b^	2.861	0.4266	6.706	**<** **0.001**	2.023	3.700
Patient‐related factors						
Age at diagnosis	−0.097	0.0533	−1.820	0.070	−0.202	0.008
Female child	−0.301	0.4442	−0.677	0.499	−1.175	0.574
Time since diagnosis	−0.023	0.0107	−2.134	**0.034**	−0.044	−0.002
Random effects						
Residual *σ* ^2^	14.885	1.502	9.910	**<** **0.001**	12.214	18.140
ICC	0.227					

*Note:* The bold value signifies the statistically significant results, with *p*‐values less than 0.05.

Abbreviations: CI, confidence interval; Est, estimations; ICC, intraclass correlation coefficient; SE, standard error.

^a^ Yes/no.

^b^ FAD‐GF score.

^c^ Leukemia versus CNS tumor.

^d^ FoP‐Q‐SF/PR‐Score.

^e^ SES according to Winkler.

Some predictors were specific to either depression or anxiety. For depressive symptoms, being a single parent/caregiver, unemployment, and higher FoP emerged as significant predictors. In contrast, for anxiety symptoms, being a female parent/caregiver was significantly associated with higher symptom levels in the final model.

The intraclass correlation coefficient (ICC) values were low for both depressive symptoms (ICC = 0.119) and anxiety symptoms (ICC = 0.227), indicating limited within‐family clustering [29].

### 3.4 Predictors of Symptom Changes From EOT to 12–18 Months During Aftercare

The GLMM results showed that parents with higher baseline anxiety and depression experienced greater symptom reductions over the 12–18‐months follow‐up period (Table S1). Conversely, a longer time since diagnosis was significantly associated with smaller symptoms reduction or even worsening. No other significant predictors were identified. Specifically, COVID‐19 was not significantly associated with either outcome. The low ICC values for both depression (ICC = 0.295) and anxiety changes (ICC = 0.165) suggest limited within‐family clustering.

## 4 Discussion

When a child has cancer, parents undergo a life‐changing experience that often leads to significant psychological distress. This study investigated the prevalence, predictors, and changes in anxiety and depressive symptoms among parents of CCS following the EOT.

An increase in distress around the EOT, followed by a significant decline, is commonly observed in earlier research [30, 31]. As time passes, parents adapt to the new normal and experience relief from the end of constant medical interventions. Our study confirmed this pattern, with the highest levels of anxiety and depression reported immediately after EOT and a notable decrease at follow up. Approximately 22 months after the child's cancer diagnosis, 34% of parents reported clinically significant anxiety symptoms, and 30% reported clinically significant depressive symptoms, which aligns with results of earlier studies [4, 8, 11]. During aftercare, 12–18 months later, the prevalence of clinically significant anxiety and depression decreased markedly to approximately 16% for both, reflecting substantial symptom remission. While prevalence rates remained higher than those reported in the general German population at both time points, and while psychological distress remains noteworthy at any stage, the significant symptom remission during aftercare is notable [32]. Our findings highlight the adaptive capacity of most parents and offer an optimistic perspective for many families facing a child's cancer disease.

Mothers consistently reported higher levels of anxiety and depression, a common finding attributed to factors like the primary caregiving role, emotional investment, professional sacrifices, accompanied by a lack of work‐life balance and changes in daily routine [9, 12, 33]. However, gender‐specific reporting styles and expression of feelings may also contribute to this gender disparity [34]. Although scores decreased for both parents, the disparity persisted, albeit on a lower level. A previous study reported that while fathers' mental health remained stable, mothers experienced a worsening of mental health up to 7 years after their child's diagnosis, highlighting gender‐specific patterns [9]. In contrast, a recent meta‐analysis of 58 studies found no significant gender‐specific differences in reported anxiety and depression [11]. It is important to note, that only 29% of participants in the included studies were fathers, and 17 of the studies focused exclusively on mothers, suggesting that fathers may be a neglected population in research on parental distress.

Gender differences were further reflected in our GLMM results at EOT, where being a female caregiver predicted higher anxiety levels in the final model. Furthermore, anxiety and depression were both associated with physical illness, psychotherapy, and lower family functioning. More time since diagnosis was linked to lower symptoms, and depression was further associated with unemployment, single parenting, and FoP. When considering the clinical implications of these findings, predictors of psychological distress can be viewed in terms of their level of influence and potential for intervention. Certain structural and demographic characteristics, like unemployment and physical illness, may represent high‐risk indicators (“red flags”), as they are relatively stable and typically beyond the individual's control. Other factors, like being a female caregiver, may function as potential risk‐factors (“yellow flags”), with their impact depending on contextual factors like coping strategies or social support, representing areas that are modifiable through interventions. Some variables may be better understood as correlates of distress rather than predictors. For instance, FoP and lower family functioning reflect emotional burden, not independent predictors. Similarly, previous psychotherapy may reflect a response to pre‐existing psychological challenges or an increased emotional awareness, rather than a predictor of distress.

In our GLMM results on longitudinal data, baseline anxiety and depression levels were key predictors, with higher levels linked to greater improvements. This finding suggests that parents with initially higher distress may experience greater potential for recovery post‐treatment. Time since diagnosis also emerged as significant, with a longer time since diagnosis being associated with smaller symptom reductions or even worsening, suggesting that the most significant psychological adaptation occurs early in the transition to survivorship, while prolonged survivorship may be associated with persistent or increasing psychological distress.

Clinical characteristics such as the child's gender, age, or type of cancer (leukemia vs. CNS tumor) did not predict parental anxiety or depression symptoms in any of the models, despite the differing prognoses associated with different cancers. Contrasting findings in earlier studies point to the possible influence of the child's age, the child's performance, and the cancer subtype [8, 9]. Notably, low ICC values indicated that individual differences between parents within families were more significant in predicting anxiety and depression than shared family experiences [29]. These findings suggest that parental emotional well‐being is primarily influenced by personal factors like the caregiving role and psychological state.

Overall, evidence suggests that time facilitates emotional recovery, with most parents experiencing a significant reduction in psychological distress over time. In our study, clinically significant anxiety and depressive symptoms decreased markedly during aftercare, underscoring many parents' capacity to recover as they adjust to life beyond their child's acute cancer treatment. However, our findings reveal that anxiety and depression levels in parents of CCS remain higher than in the general population, even long after EOT. This highlights the importance of recognizing that while time aids recovery, additional factors like pre‐existing psychological vulnerabilities, caregiving roles, and family dynamics likely play a crucial role in shaping long‐term mental health outcomes.

## 5 Implications

Our findings underscore the critical need for early and ongoing psychological screening for at‐risk parents in clinical settings. This screening should be complemented by tailored psychological support to improve long‐term mental health outcomes. Parents closer to the time of their child's diagnosis may particularly benefit from timely interventions. Currently, psychological services vary widely across pediatric oncology centers and are often limited to the treatment phase. After treatment, parents are frequently left responsible for seeking these services themselves, as they are too rarely actively offered [35]. In Germany, the establishment of the nationwide S3 guideline for psychosocial care in pediatric oncology and hematology is a step in the right direction [36]. However, consistent implementation is crucial to ensure that parents who may require psychological support, even years after their child's treatment, receive adequate care.

Research on long‐term anxiety and depression among parents of children with cancer remains limited, highlighting the need for extends follow‐up studies to better understand distress trajectories over time. Moreover, as fathers of children with cancer are often a neglected population in research, future work should also prioritize understanding their specific experiences of distress [11]. In addition, parents of children with cancer often identify religion and spirituality as important coping resources that enhance resilience [37, 38]. Future studies are needed that investigate the role of these factors in mitigating anxiety and depression among parents of CCS. Lastly, targeted interventions should be developed for high‐risk groups, including mothers, unemployed parents, and single parents.

## 6 Limitations

Several limitations should be considered when interpreting our results. First, this study is a secondary analysis of data from a larger project focused on rehabilitation, which prevented a non‐responder analysis and may affect the generalizability of the findings. The exclusion criteria and voluntary participation introduce a potential selection bias, as the study design does not provide a comprehensive overview of the entire population, and it is possible that only families less burdened by their circumstances ultimately participated. Additionally, some patients may have received maintenance therapy during the study period, which could have influenced the results.

The step‐down stepwise procedure enhances model interpretability and reduces complexity by focusing on significant predictors [23]. However, it increases the risk of overfitting and may lead to multiple hypothesis testing issues, potentially reducing model robustness [39]. Clinically relevant variables could also be overlooked if they don't meet inclusion thresholds. Despite these limitations, the exploratory nature of this study prioritized gaining an initial overview of key factors in parental psychological distress, providing valuable insights into the psychological burden faced by parents of childhood cancer survivors. Furthermore, the comparatively large sample size, inclusion of both mothers and fathers, and use of validated questionnaires strengthen the reliability of our findings in this research field.

## 7 Conclusion

About one‐third of parents in our sample experienced clinically relevant symptoms of depression and anxiety after the EOT. Our findings demonstrated a marked decrease in these symptoms during aftercare, highlighting the potential for emotional recovery for many parents. These results emphasize the importance of providing early and ongoing psychosocial screening and needs‐based support for parents of CCS. We identified both modifiable and structural predictors of parental distress. Certain demographic and structural characteristics, such as parental illness or unemployment, may serve as high‐risk indicators. In contrast, factors such as being a mother may function as moderate‐risk indicators, with distress being influenced by contextual factors like coping resources. Some factors, such as FoP and family functioning, were more reflective of correlates of distress rather than predictors, suggesting that they could be specifically targeted by health care providers within psychosocial interactions. Overall, integrating psychological support into routine cancer care and after care is crucial to address the psychological burden experienced by parents of CCS. Future research is needed to assess long‐term psychological distress and adjustment during survivorship or palliation.

## Author Contributions

C.B. served as the principal investigator for the study. The study concept and design were developed by C.B., L.I., M.L.N., and H.K. Material preparation and data collection were carried out by L.I., M.L.N., V.P., K.A.K., D.K., G.E., and S.R. H.K. was responsible for data analysis, interpretation, and drafting the initial manuscript. All authors reviewed and approved the final manuscript.

## Ethics Statement

This study was approved by the Ethics Committee of the Medical Chamber of Hamburg (Reference Number PV5277).

## Consent

Informed consent was obtained from all families involved in the study. As the data are anonymized, consent for publication was not required.

## Conflicts of Interest

The authors declare no conflicts of interest.

## Large Language Models

ChatGPT was used to assist with translations, grammar, and language corrections [40].

## Supporting information

Table S1

## Data Availability

The data supporting the findings of this study can be obtained from the corresponding author upon reasonable request and after correspondence with the data protection manager of the institution.
