# Supplementary material for: Beyond Treatment: Prevalence, Predictors, and Changes in Anxiety and Depression Among Parents of Childhood Cancer Survivors
Source: Psychooncology. 2025 Mar 2;34(3):e70115. doi: 10.1002/pon.70115 (PMC11872680; doi:10.1002/pon.70115)
Supplement: Supplementary file 1 — Table S1 [file PON-34-e70115-s001.docx]

| **Supplementary Table 1: Mixed Model Estimates for Predicting Changes of Anxiety and Depression in Parents of Childhood Cancer Survivors Within 12–18 Months Post-Treatment** | | | | | |  |
| --- | --- | --- | --- | --- | --- | --- |
| **Supplementary Table 1a: Predictors of Depression Change in Parents Within 12–18 Months Post-Treatment** | | | | | | |
| **Factor** | **Est.** | **SE** | **t** | **p** | **95%CI** |  |
|  |  |  |  |  | **Lower** | **Upper** |
| **Intercept** | -0.689 | 1.114 | -0.618 | 0.537 | -2.882 | 1.505 |
| **Parent-Related Factors** |  |  |  |  |  |  |
| Depressive symptoms at baseline (PHQ-9) † | -0.471 | 0.052 | -9.050 | **<0.001** | -0.573 | -0.368 |
| FoP at baseline ‡ | 0.022 | 0.028 | 0.777 | 0.438 | -0.033 | 0.077 |
| Participation in psychotherapy § | 0.495 | 0.469 | 1.057 | 0.291 | -0.427 | 1.418 |
| **Family-Related Factors** |  |  |  |  |  |  |
| Assessment during COVID-19 pandemic § | -0.533 | 0.685 | -0.777 | 0.438 | -1.887 | 0.822 |
| Family functioning (FAD-GF) | 0.626 | 0.479 | 1.308 | 0.192 | -0.316 | 1.568 |
| **Patient-Related Factors** |  |  |  |  |  |  |
| Diagnosis (Leukemia) ¶ | 0.194 | 0.500 | 0.389 | 0.698 | -0.793 | 1.181 |
| Female child | -0.093 | 0.396 | -0.234 | 0.815 | -0.875 | 0.690 |
| Time since diagnosis (Months) | 0.031 | 0.012 | 2.590 | **0.010** | 0.007 | 0.054 |
| **Random Effects** |  |  |  |  |  |  |
| Residual σ² | 8.930 | 1.193 | 7.487 | **<0.001** | 6.873 | 11.603 |
| ICC | 0.295 |  |  |  |  |  |
| † PHQ-9-Score at baseline; ‡ FoP-Q-SF/PR-Score at baseline; § yes/no; ¶ Leukemia vs. CNS tumor | | | | |  |  |
| CI: Confidence interval; Est: Estimations; ICC: Intraclass correlation coefficient; SE: Standard error | | | | |  |  |
|  |  |  |  |  |  |  |
| **Supplementary Table 1b: Predictors of Anxiety Change in Parents Within 12–18 Months Post-Treatment** | | | | | | |
| **Factor** | **Est.** | **SE** | **t** | **p** | **95%CI** |  |
|  |  |  |  |  | **Lower** | **Upper** |
| **Intercept** | 1.089 | 1.480 | 0.736 | 0.463 | -1.828 | 4.006 |
| **Parent-Related Factors** |  |  |  |  |  |  |
| Age | -0.028 | 0.036 | -0.785 | 0.433 | -0.098 | 0.042 |
| Anxiety at baseline † | -0.484 | 0.045 | -10.724 | **<0.001** | -0.573 | -0.395 |
| Gainfully employed ‡ | -0.689 | 0.541 | -1.274 | 0.204 | -1.755 | 0.377 |
| Serious physical illness ‡ | 1.196 | 0.772 | 1.550 | 0.122 | -0.324 | 2.716 |
| **Family-Related Factors** |  |  |  |  |  |  |
| Assessment during COVID-19 pandemic ‡ | -0.566 | 0.607 | -0.932 | 0.353 | -1.767 | 0.635 |
| Family functioning at baseline § | 0.780 | 0.449 | 1.739 | 0.083 | -0.104 | 1.664 |
| **Patient-Related Factors** |  |  |  |  |  |  |
| Age at Diagnosis | 0.081 | 0.061 | 1.342 | 0.181 | -0.038 | 0.201 |
| Time since Diagnosis (Months) | 0.030 | 0.011 | 2.747 | **0.007** | 0.009 | 0.052 |
| **Random Effects** |  |  |  |  |  |  |
| Residual σ² | 8.950 | 1.243 | 7.202 | <0.001 | 6.818 | 11.749 |
| **ICC** | 0.165 |  |  |  |  |  |
| † GAD-7-Scores at baseline; ‡ yes/no; § FAD-GF Scores at baseline | | |  |  |  |  |
| CI: Confidence interval; Est: Estimations; ICC: Intraclass correlation coefficient; SE: Standard error | | | | |  |  |
|  |  |  |  |  |  |  |
